# Author Correction: Host-associated niche metabolism controls enteric infection through fine-tuning the regulation of type 3 secretion

**DOI:** 10.1038/s41467-018-07612-0

**Published:** 2018-11-29

**Authors:** James P. R. Connolly, Sabrina L. Slater, Nicky O’Boyle, Robert J. Goldstone, Valerie F. Crepin, David Ruano-Gallego, Pawel Herzyk, David G. E. Smith, Gillian R. Douce, Gad Frankel, Andrew J. Roe

**Affiliations:** 1 Institute of Infection, Immunity and Inflammation, University of Glasgow, Glasgow, G12 8TA UK; 2 MRC Centre for Molecular Bacteriology and Infection, Department of Life Sciences, Imperial College, London, SW7 2AZ UK; 3 School of Life Science, Heriot-Watt University, Edinburgh, EH14 4AS UK; 4 Glasgow Polyomics, Wolfson Wohl Cancer Research Centre, University of Glasgow, Garscube Estate, Glasgow, G61 1QH UK

Correction to: *Nature Communications*. doi: 10.1038/s41467-018-06701-4; published online 10 Oct 2018

The original version of this Article contained an error in the spelling of the author David Ruano-Gallego, which was incorrectly given as David R. Gallego. This has now been corrected in both the PDF and HTML versions of the Article.

