# Supplementary material for: Host-associated niche metabolism controls enteric infection through fine-tuning the regulation of type 3 secretion
Source: Nat Commun. 2018 Oct 10;9:4187. doi: 10.1038/s41467-018-06701-4 (PMC6180029; doi:10.1038/s41467-018-06701-4)
Supplement: Supplementary file 1 — Supplementary Information [file 41467_2018_6701_MOESM1_ESM.pdf]

Host-associated niche metabolism controls enteric infection through fine-tuning the regulation of type 3 secretion

Connolly *et al.*

Supplementary Information

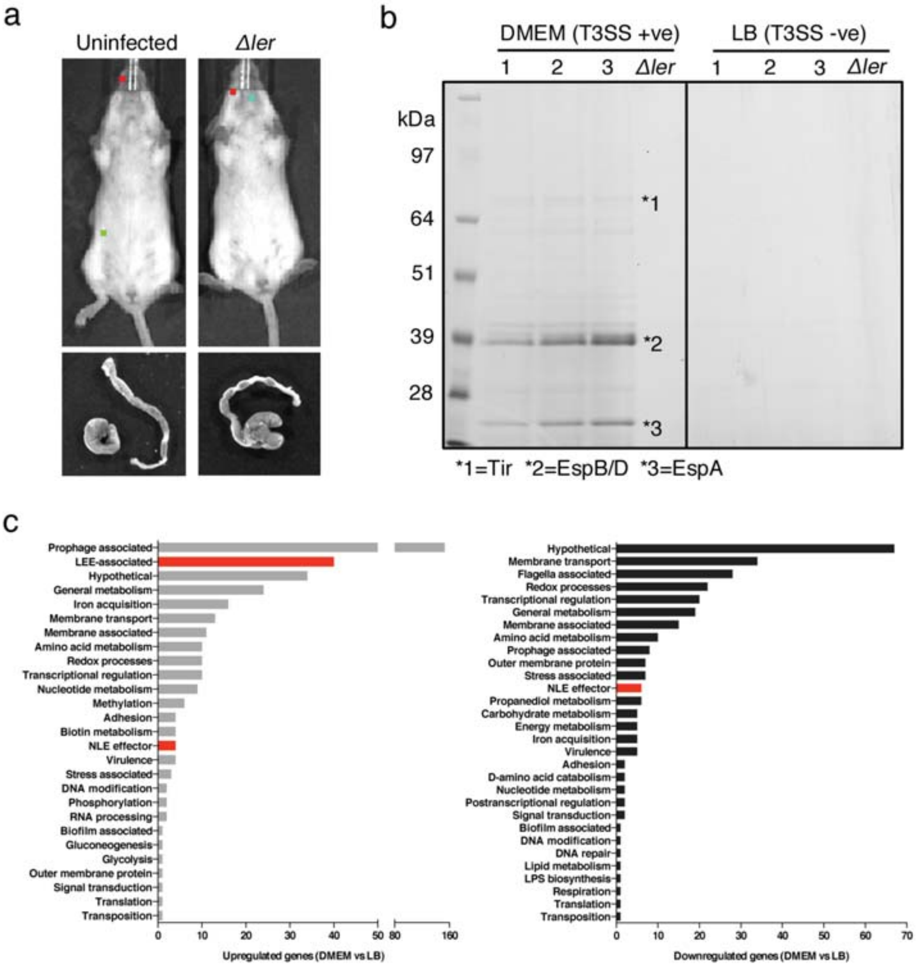

Supplementary Figure 1. Experimental controls for *in vivo* RNA-seq analysis. **a** IVIS images of control mice used for *in vivo* RNA-seq experiments. Images show a representative mouse from the uninfected and Δ*ler* groups as well as corresponding intestine dissections. **b** SDS-PAGE gel profiling of secreted proteins from *C. rodentium* wild type cells used for *in vitro* RNA-seq. The presence of T3SS- associated secreted proteins (labelled as *1-3) indicates the status of the conditions used (DMEM/T3SS +ve or LB/T3SS -ve respectively). Δ*ler* was used as a T3SS deficient control for the secreted profiling. **c** RNA-seq analysis of *in vitro* control conditions used in this study (DMEM vs LB T3SS). The grouping of respective functional groups are listed and LEE/NLE genes are indicated in red.

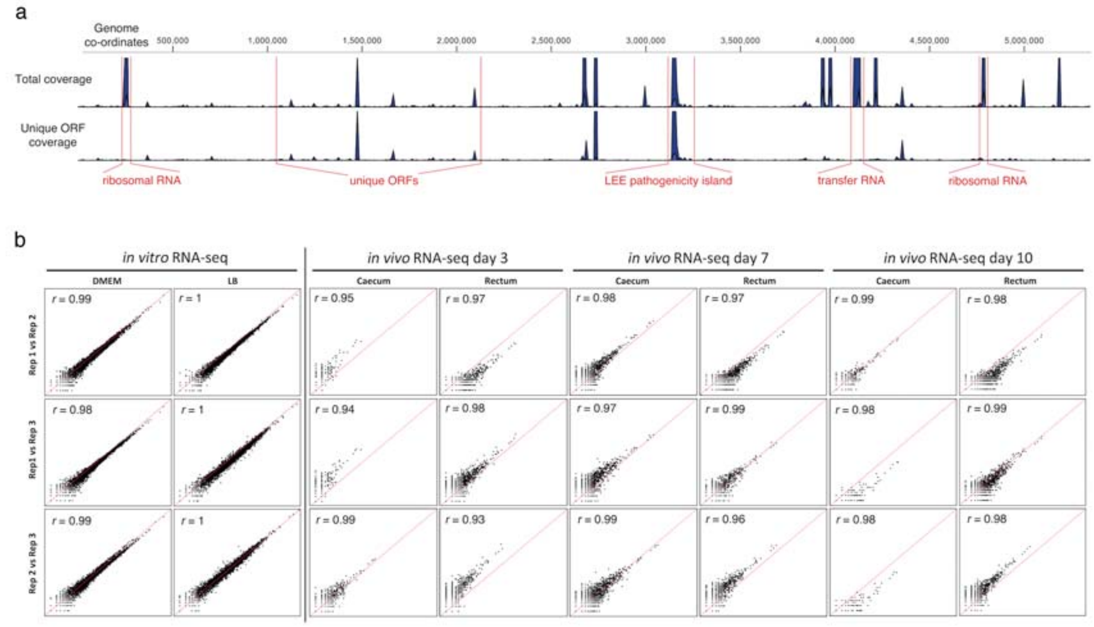

Supplementary Figure 2. Mapping of *in vivo* RNA-seq data. a Representative coverage plot of mapped RNA-seq reads illustrating the total and unique read alignment. Selected examples of reads mapped to tRNA, rRNAs (total coverage) or unique coding regions are indicated in red. **b** Normalized read counts (log_2 transformed) of genes identified by *in vitro* and *in vivo* RNA-seq in all samples tested. Replicates were compared pairwise with the Pearson correlation coefficient (*r*) indicated above.

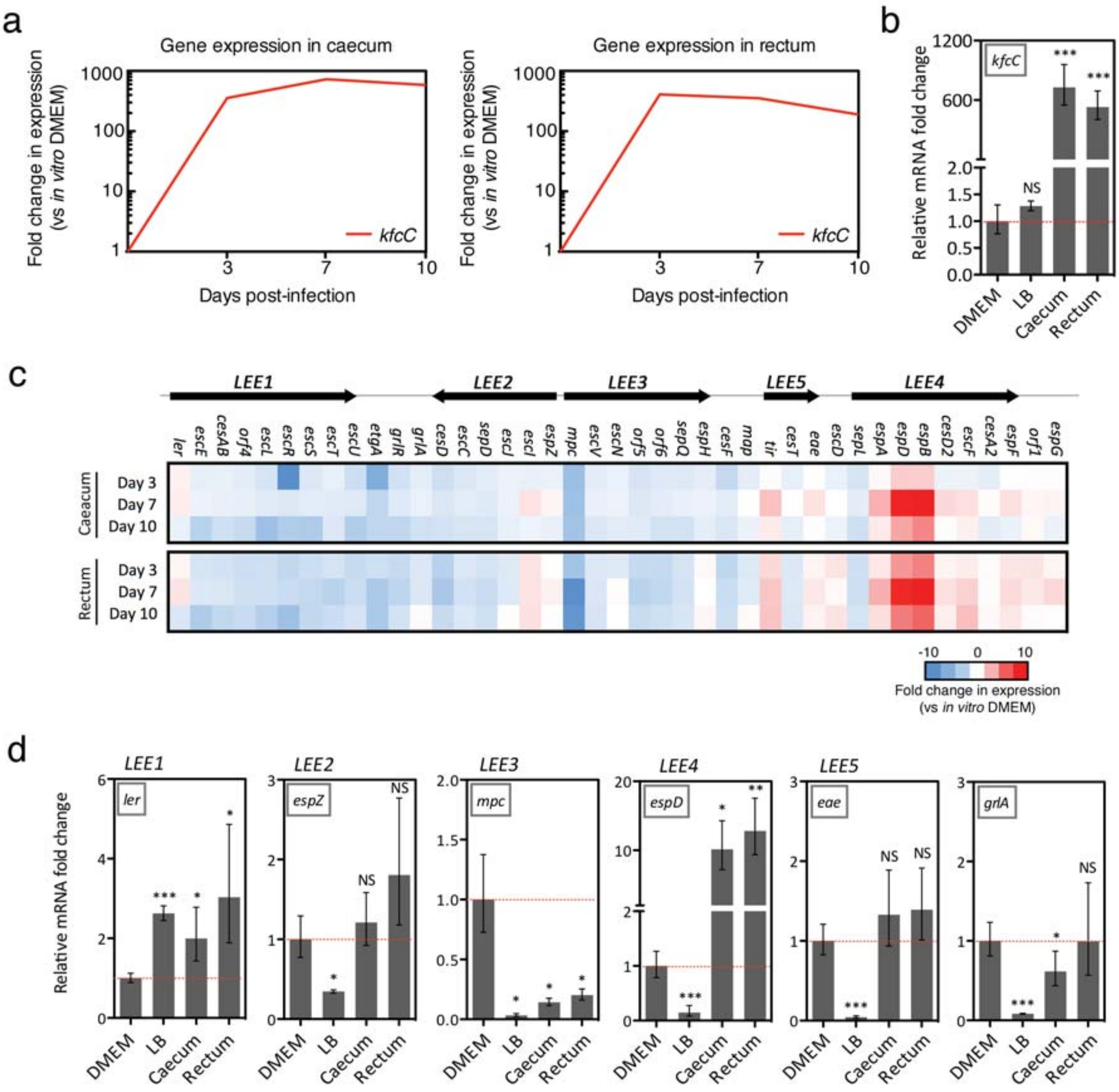

Supplementary Figure 3. Validation of virulence factor expression *in vivo*. **a** Line plots illustrating expression dynamics of *kfcC* during infection versus DMEM. **b** qRT-PCR validation of *kfcC* expression *in vivo*. *** and NS denote P ≤ 0.001 and not significant respectively as compared to baseline expression in DMEM (red dotted line). **c** Heatmap of LEE pathogenicity island fold-change in expression (EDGE test) versus growth in DMEM (T3SS +ve). All ORFs and respective LEE operons are labelled above. Each node indicates the mean of three biological replicates. The color scale is indicated below. **d** qRT-PCR validation of LEE expression *in vivo*. Selected genes from each LEE operon were analyzed. *, **, *** and NS denote P ≤ 0.05, P ≤ 0.01, P ≤ 0.001 and not significant respectively as compared to baseline expression in DMEM (red dotted line).

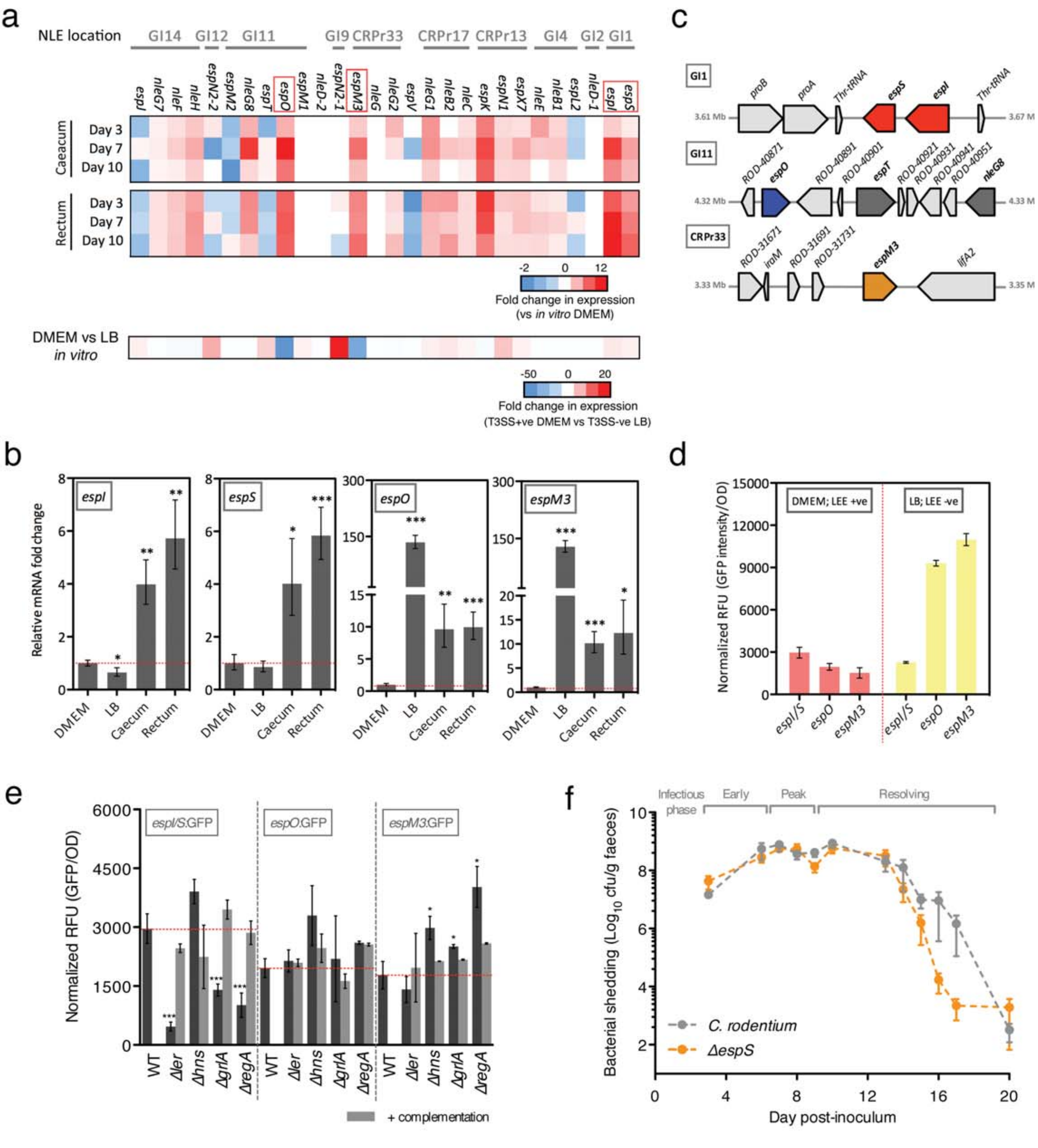

Supplementary Figure 4. NLE expression and regulation in *C. rodentium*. **a** Heatmaps of NLE gene fold-changes in expression *in vivo* (EDGE test) versus growth in DMEM (T3SS +ve). All NLEs and respective genomic location are labelled above. The second heatmap (below) indicates NLE fold change in expression in DMEM versus growth in LB, highlighting the heterogeneous nature of NLE expression. Each node indicates the mean of three biological replicates. The color scale for each heatmap is indicated below. **b** qRT-PCR validation of NLE expression *in vivo*. *, ** and *** denote P ≤ 0.05, P ≤ 0.01 and P ≤ 0.001 respectively. **c** Genomic context of the most highly expressed NLEs *in vivo* identified by *in vivo* RNA-seq. **d** Reporter activity of p*espI/S*:GFP, p*espO*:GFP and p*espM3*:GFP in *C. rodentium* cultured in DMEM or LB to validate environmentally-dependent differential regulation of individual NLEs. Expression was measured as GFP/OD_{600} from the mean of three biological replicates (+SEM). **e** Reporter activity of p*espI/S*:GFP, pespO:GFP and p*espM3*:GFP in defined regulator mutants (Δ*ler*, Δ*hns*, Δ*grlA* and Δ*regA*). The red dotted lines indicate the base line expression of each reporter. Relative fluorescence units (RFU) were measured as GFP/OD_{600} and representing the mean of three biological replicates ±SEM. * and *** denote P ≤ 0.05 and P ≤ 0.001 respectively compared to the wild type (Students f-test). **f** Colonization of the murine host by wild type *C. rodentium* or Δ*espS*. Each data point represents the mean bacterial load (±SEM) in feces of infected mice (groups of n=5 mice; CFU/g). The infectious phase of each sampling point is indicated above the graph.

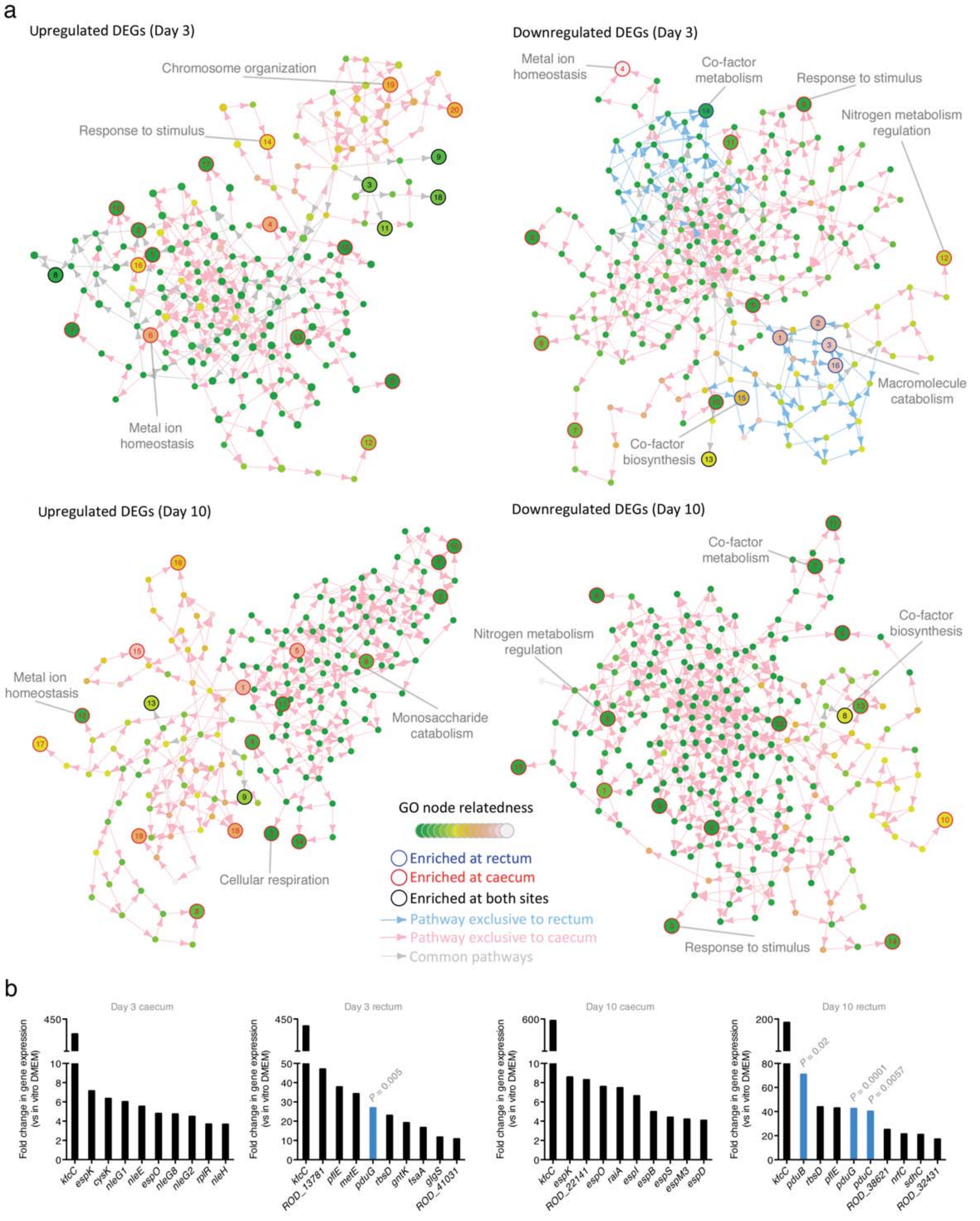

Supplementary Figure 5. Functional clustering of DEGs identified by *in vivo* RNA-seq. **a** Network graphs illustrating the clustering and identification of GO terms in vivo at the early and late time- points (days 3 and 10). Networks were generated for both upregulated and downregulated genes individually. Colored nodes represent GO terms associated with the DEGs *in vivo* and the arrows indicate relatedness to one another by distance. Significantly enriched GO terms (P ≤ 0.05) were assigned a numbered node and each node is color coded to represent enrichment of a biological function at the caecum (red), the rectum (blue) or at both sites (black). Selected GO terms are labelled in grey. **b** Top DEGs identified according to fold increase in expression at early and late infection. *pdu* genes are highlighted in blue along with the relative P-value above. Each bar represents 3 biological replicates (EDGE test).

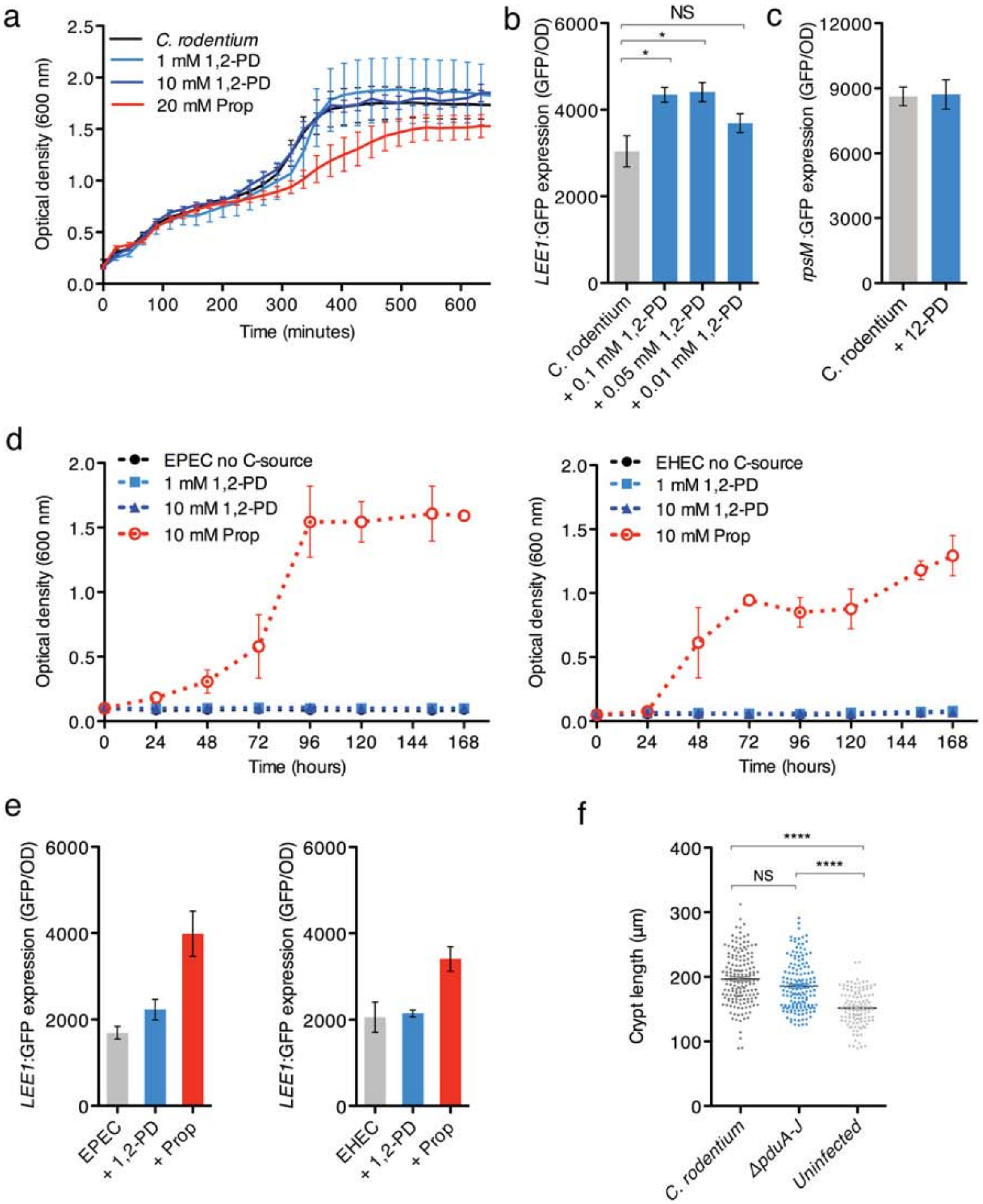

Supplementary Figure 6. 1,2-propanediol metabolism and virulence regulation in A/E pathotypes. **a** Growth of *C. rodentium* in DMEM supplemented with 1,2-PD or Prop. Data represents the mean optical density (600nm) of three biological replicates (±SEM). **b** *LEE1*:GFP reporter activity in DMEM supplemented with a gradient of 1,2-PD concentrations (0.1 mM, 0.05 mM and 0.01 mM). *LEE1* expression was measured as GFP/OD600 Data represents the mean of three biological replicates ±SEM. * and NS denote P ≤ 0.05 and not significant respectively (Students t-test). **c** Reporter assay in DMEM with and without 1,2-PD supplementation (1 mM) using *C. rodentium* transformed with p*rpsM*. Relative fluorescence units (RFU) were measured as GFP/OD_{600} and the data represents the mean of three biological replicates ±SEM. **d** Growth of EPEC and EHEC with 1,2-PD or Prop as a sole carbon source. Data represents the mean optical density (600nm) of three biological replicates (±SEM). **e** p*rpsM*:GFP reporter activity of EPEC and EHEC in DMEM supplemented with 1,2-PD or Prop. LEE1 expression was measured as RFU (GFP/OD_{600}) Data represents the mean of three biological replicates ±SEM. **f** Enumeration of colonic epithelial cell crypt length (pm) from mice infected with either wild type *C. rodentium*, Δ*pduA-J* or uninfected controls. Sampling was carried out at peak infection (day eight PI). For quantification, **** and NS denote P ≤ 0.0001 or not significant (1-way ANOVA with Bonferroni multiple correction test).

Supplementary Table 1. Bacterial strains used in this study

Name	Description	Reference
C. rodentium	*C. rodentium* ICC169; O152 serotype; Nalidixin resistant	Petty *et al*. 2010
C. rodentium LUX	C. rodentium ICC169 LUX marked at 16S locus; Erythromycin resistant	This study
Δler	*C. rodentium* ICC169 *ler* deletion mutant; Kanamycin resistant	This study
Δler LUX	*C. rodentium* ICC169 luminescent *ler* deletion mutant; Erythromycin/Kanamycin resistant	This study
ΔgrlA	*C. rodentium* ICC169 *grlA* deletion mutant; Kanamycin resistant	This study
ΔregA	*C. rodentium* ICC169 *regA* deletion mutant; Kanamycin resistant	This study
Δhns	*C. rodentium* ICC169 *hns* deletion mutant; Kanamycin resistant	This study
ΔespS	*C. rodentium* ICC169 *espS* deletion mutant;	This study
ΔpduA-J	*C. rodentium* ICC169 *pduA-J* deletion mutant; Kanamycin resistant	This study
EHEC	*E. coli* TUV93-0	Campellone *et al*., 2002
EPEC	*E. coli* E2348/69	Iguchi *et al*., 2009
ICC1370	*C. rodentium* constitutive-Pler luminescent; Nalidixin/Kanamycin resistant	Mullineaux-Sanders *et al*. 2017
ICC1370 *ΔpduA-J*	*C. rodentium* constitutive-Pler luminescent; Nalidixin/Kanamycin resistant	This study

Supplementary Table 2. Plasmids used in this study

**Name**	**Description**	**Reference**
pKD46	Red recombinase system; arabinose inducible; 30°C permissive; ampicillin resistant	Datsenko and Wanner 2000
pCP20	FLP recombinase system; 30°C permissive; ampicillin resistant	Datsenko and Wanner 2000
pKD4	Kanamycin-FRT cassette template plasmid; *oriRγ* replicon; Kanamycin resistant	Datsenko and Wanner 2000
pSIM18	Temperature inducible Red recombinase system; 42°C inducible; Hygromycin resistant	Chan *et al.* 2007
pBAD:*pduA-J*	*pduA-J* cloned into pBAD18; arabinose inducible; Ampicillin resistant	This study
pACBSR	Receiver plasmid. *p15A* ori, *PBAD,* I-SceI, endonuclease and λ-red genes; chloramophenicol re	Herring *et al.,* 2003
pSEVA612S	*R6K* ori, *oriT*, I-SceI sites flanking multiple cloning site; gentamicin resistant	Martinez-Garcia *et al.,* 2015
pSEVA612SΔesp S	Donor plasmid. pSEVA612S backbone containing 300 bp homology regions flanking *espS*	This study
pAJR70	pACYC184 containing single *BamhI/KpnI* cloning site in-frame with eGFP; Chloramphenicol res	Roe *et al*. 2003
p*rpsM*:GFP	pAJR70 containing *rpsM* promoter fused to *gfp+*; Chloramphenicol resistant	Roe Lab inventory
p*espI/S*:GFP	*C. rodentium espI/S* promoter region cloned in-frame into pAJR70; Chloramphenicol resistant	This study
p*espO*:GFP	*C. rodentium espO* promoter region cloned in-frame into pAJR70; Chloramphenicol resistant	This study
p*espM3*:GFP	*C. rodentium espM3* promoter region cloned in-frame into pAJR70; Chloramphenicol resistant	This study
Eh*LEE1*:GFP	EHEC *LEE1* promoter region cloned in-frame into pAJR70; Chloramphenicol resistant	Roe *et al*. 2003
Ep*LEE1*:GFP	EPEC *LEE1* promoter region cloned in-frame into pAJR70; Chloramphenicol resistant	This study
Cr*LEE1*:GFP	*C. rodentium LEE1* promoter region cloned in-frame into pAJR70; Chloramphenicol resistant	This study
pWSK:*ler*	*ler* cloned into pWSK29; IPTG inducible; Ampicillin resistant	This study
pWSK:*grlA*	*grlA* cloned into pWSK29; IPTG inducible; Ampicillin resistant	This study
pWSK:*hns*	*hns* cloned into pWSK29; IPTG inducible; Ampicillin resistant	This study
pWSK:*regA*	*regA* cloned into pWSK29; IPTG inducible; Ampicillin resistant	This study

Supplementary Table 3. Oligonucleotide primers used in this study

Name	Description	Sequence
ler KO F	Lambda Red knockout primer	TGACCCATCCATGTAAGGATGAGCTTGTTAATATCTTAATATATAAAAGTGTGTAGGCTGGAGCTGCTTC
ler KO R	Lambda Red knockout primer	TATTATTTCATCTTCCAGTTCAGTTATCGTTATCATTTAATTATTTCATGCATATGAATATCCTCCTTAG
ler check F	Lambda Red knockout check primer	CCATCCATGTAAGGATGAGC
ler check R	Lambda Red knockout check primer	TGCAATGAGCAGTTCCTTTG
grlA KO F	Lambda Red knockout primer	TTTATTGTTTTTTTGTGTTAATTTTTTATCAAATAAAAAGAACATGGAAAGTGTAGGCTGGAGCTGCTTC
grlA KO R	Lambda Red knockout primer	AATAGGGATCTTTTTTTTATTATTCTATAAACTTACTAAGAAAGCTACGTCATATGAATATCCTCCTTAG
grlA check F	Lambda Red knockout check primer	GGCGAACAGAATAAACTCAG
grlA check R	Lambda Red knockout check primer	ATGTCAGATTTATCGAACCG
hns KO F	Lambda Red knockout primer	CATACATTATTTTTATTAATCGCCCCTTTTAAAACACCGGACAACATACTGTGTAGGCTGGAGCTGCTTC
hns KO R	Lambda Red knockout primer	AACCTGTTCAGGTAGCAGGCTCTACCAGAGAAAATCAGAGTTAATACAAGCATATGAATATCCTCCTTAG
hns check F	Lambda Red knockout check primer	ACATTATTTTTATTAATCGCCC
hns check R	Lambda Red knockout check primer	TAGCAGGCTCTACCAGAGAA
regA KO F	Lambda Red knockout primer	TAGCTGATAACCCATGAAATTATATTCATACAAAGCATGGGGGTGACTGTGTGTAGGCTGGAGCTGCTTC
regA KO R	Lambda Red knockout primer	GCTAAACGTATCAGAACATAACCTGAAGAACTGCTAACTACCCGGATCGTCATATGAATATCCTCCTTAG
regA check F	Lambda Red knockout check primer	AGCTGATAACCCATGAAATTA
regA check R	Lambda Red knockout check primer	CGTATCAGAACATAACCTGAAG
ler pWSK F	Complementation plasmid primer	CCCTCTAGACCATCCATGTAAGGATGAG
ler pWSK R	Complementation plasmid primer	CCCGGATCCTTAAATGTTATTCAGAG
grlA pWSK F	Complementation plasmid primer	CCGAGCTCAAGAACATGGAAAATGG
grlA pWSK R	Complementation plasmid primer	CCGGATCCCTAACTCTCCTTTTTTCGTC
hns pWSK F	Complementation plasmid primer	CCGAGCTCATAAGTTTGAGATTACTACA
hns pWSK R	Complementation plasmid primer	CCGGATCCTTATTCCTTGATCAGGAAATC
regA pWSK F	Complementation plasmid primer	CCGAGCTCCAAAGCATGGGGGTGACTGT
regA pWSK R	Complementation plasmid primer	CCGGATCCTCACGGCTGTTTCTTCACT
pduA-J KO F	Lambda Red knockout primer	TCGCTTCTTTTTATAGTCAGCAATATCGGGACAATCCAGGCGAGGTCTTTGTGTAGGCTGGAGCTGCTTC
pduA-J KO R	Lambda Red knockout primer	TTCAAGTAATCCCAGTGATTGCTTCACGCTGTGCTCCTTTTCGACAGTACCATATGAATATCCTCCTTAG
pduA-J check F	Lambda Red knockout check primer	TTTTAGCCGCAGCGTTAAG
pduA-J check R	Lambda Red knockout check primer	ATGGCTAATGCCAGACCACT
pBAD18 F	NEBuilder custom assembly primer	CTAGCGAATTCGAGCTCG
pBAD18 R	NEBuilder custom assembly primer	CCCAAAAAAACGGGTATGG
pduA-J fragment 1 F	NEBuilder custom assembly primer	GTTTCTCCATACCCGTTTTTTTGGGAATCCAGGCGAGGTCTTTATG
pduA-J fragment 1 R	NEBuilder custom assembly primer	GAACGATAAGGACGCAGGGCGTTGTAAATCTC
pduA-J fragment 2 F	NEBuilder custom assembly primer	TACAACGCCCTGCGTCCTTATCGTTCAACGAAAGAG
pduA-J fragment 2 R	NEBuilder custom assembly primer	CGGGTACCGAGCTCGAATTCGCTAGACGCTGTGCTCCTTTTCG
espS check F	Triparental conjugation	ATCCCGTTAATAAGCAGAGAGGTAAAAGAG
espS check R	Triparental conjugation	TTATTCATTTAAAAATAATAGCTGAAATGG
espI pAJR70 F	promoter-eGFP fusion cloning	CCGGATCCCACCAAATACAATTAATTGAT
espI pAJR70 R	promoter-eGFP fusion cloning	CCGGTACCGGTGATTCCGGAATGTATGTT
espO pAJR70 F	promoter-eGFP fusion cloning	CCGGATCCGGACAAAAATATAAACTGCTT
espO pAJR70 R	promoter-eGFP fusion cloning	CCGGTACCAGCACGTGAAAATATATTTCT
espM3 pAJR70 F	promoter-eGFP fusion cloning	CCGGATCCTACCCCCTGCAATAATGGTAT
espM3 pAJR70 R	promoter-eGFP fusion cloning	CCGGTACCAGAATTAAAAGATAAACCTTG
Cr LEE1 pAJR70 F	promoter-eGFP fusion cloning	CCGGATCCGGCAAATCGGGTACGCGATCT
Cr LEE1 pAJR70 R	promoter-eGFP fusion cloning	CCGGTACCCTCAATCAGCTGAATGTATGG
Ep LEE1 pAJR70 F	promoter-eGFP fusion cloning	CCGGATCCCGAATTAAGTAGAGTATAGTG
Ep LEE1 pAJR70 R	promoter-eGFP fusion cloning	CCGGTACCCTCTATAAGCTGAATGTATGG
pAJR70 check F	promoter-eGFP fusion check primer	ATGGTGCATGCAAGGAGAT
pAJR70 check R	promoter-eGFP fusion check primer	CAGATGAACTTCAGGGTCAG
ler qPCR F	qRT-PCR primer	GAGCAGGAGATTCAAACTGT
ler qPCR R	qRT-PCR primer	TACCCCAGTTCTTGTAAGGT
grlA qPCR F	qRT-PCR primer	GTAAATTGCAGGAGAAATGG
grlA qPCR R	qRT-PCR primer	AATAAATGACGCTCTCCTCA
espD qPCR F	qRT-PCR primer	GCTACGGCTATTTCAGGTAT
espD qPCR R	qRT-PCR primer	GATGGGGCAAAAGATTTAAC
eae qPCR F	qRT-PCR primer	TTATTCATGGTTTTTGCACC
eae qPCR R	qRT-PCR primer	AAAACAATCCTAAACCAGCA
mpc qPCR F	qRT-PCR primer	TTTTATTGAGGTAATTGGTGG
mpc qPCR R	qRT-PCR primer	AGGGCACTGAAGAAAGAAA
espZ qPCR F	qRT-PCR primer	CTGCAATAAATGGAAATGGT

espZ qPCR R	qRT-PCR primer	AAAACTCTGACATTGCGACT
gapA qPCR F	qRT-PCR primer	GTAGGTATCAACGGTTTTGG
gapA qPCR R	qRT-PCR primer	CGTCTAACAGGTCGTTGATT
espI qPCR F	qRT-PCR primer	TGAACATTCAACCGAACATA
espI qPCR R	qRT-PCR primer	GCTAGAGACAGGCACTTGTT
espS qPCR F	qRT-PCR primer	TAGGTATTCTTGGCAAAAGG
espS qPCR R	qRT-PCR primer	TCCTACGGATTTTTCACCTA
espO qPCR F	qRT-PCR primer	GGCCTGTAATTGATAAACCA
espO qPCR R	qRT-PCR primer	TGGTCCTTCTGTTTATGACC
espM3 qPCR F	qRT-PCR primer	GTTGTAAAATGCTCAATGGG
espM3 qPCR R	qRT-PCR primer	TTTAGTACTCTGCCGGGTTA
kfcC qPCR F	qRT-PCR primer	ACTGTTAATTTTATGGGGGC
kfcC qPCR R	qRT-PCR primer	GTTCCTAAGTCAACACTGGC

Supplementary references

Campellone, K.G., Giese, A., Tipper, D.J., and Leong, J.M. (2002). A tyrosine-phosphorylated 12-amino-acid sequence of enteropathogenic Escherichia coli Tir binds the host adaptor protein Nck and is required for Nck localization to actin pedestals. Mol. Microbiol. *43*, 1227– 1241.

Iguchi, A., Thomson, N.R., Ogura, Y., Saunders, D., Ooka, T., Henderson, I.R., Harris, D., Asadulghani, M., Kurokawa, K., Dean, P., et al. (2009). Complete genome sequence and comparative genome analysis of enteropathogenic Escherichia coli O127:H6 strain E2348/69. J. Bacteriol. *191*, 347–354.
