# Supplementary material for: Host-associated niche metabolism controls enteric infection through fine-tuning the regulation of type 3 secretion
Source: Nat Commun. 2018 Oct 10;9:4187. doi: 10.1038/s41467-018-06701-4 (PMC6180029; doi:10.1038/s41467-018-06701-4)
Supplement: Supplementary file 3 — Description of Additional Supplementary Files [file 41467_2018_6701_MOESM3_ESM.pdf]

Description of Additional Supplementary Files

File Name: Supplementary Data 1
Description: Table of the mapping statistics from *in vivo* and *in vitro* RNA-seq experiments.

File Name: Supplementary Data 2
Description: Edge test results for RNA-seq data comparisons between *in vivo* and *in vitro* samples.

File Name: Supplementary Data 3
Description: Edge test results for RNA-seq data comparisons between *in vitro* cultured samples.

File Name: Supplementary Data 4
Description: Gene ontology (GO) analysis of DEGs identified by RNA-seq.
